# Heart rate variability biofeedback in patients with functional dizziness

**DOI:** 10.1007/s00415-025-12988-3

**Published:** 2025-03-12

**Authors:** Erik Simon, Ana Isabel Penzlin, Martin Arndt, Timo Siepmann, Kristian Barlinn

**Affiliations:** 1 Department of Neurology, University Hospital Carl Gustav Carus, Technische Universität Dresden, Dresden, Germany; 2 Department of Neurology, Rhön Klinikum, Campus Bad Neustadt, Bad Neustadt a.d. Saale, Germany

**Keywords:** HRV, RMSSD, PPPD, Parasympathetic, Depression

## Abstract

**Background:**

Functional dizziness is one of the most common causes of chronic dizziness. Associated psychiatric diseases such as depression and anxiety lead to significant impairment, possibly due to autonomic nervous system imbalance. We investigated whether heart rate variability (HRV) biofeedback can modulate autonomic function in patients with functional dizziness.

**Methods:**

We performed a randomized controlled study in 24 patients diagnosed functional dizziness for the first time. Patients received six 20 min sessions of HRV biofeedback or no intervention. We assessed HRV (time and frequency domains), sympathetic vasomotor function, sympathetic skin response and psychometric assessments at baseline, immediately post-intervention (or control period) and another 3 and 6 weeks later.

**Results:**

Patients in the HRV biofeedback group showed improved cardiac autonomic function with elevated HRV time-dependent parameters immediately post-intervention [Root Mean Square of Successive Differences (RMSSD): 71.2 ms ± 38 ms vs. 38.2 ms ± 18.5 ms, p = 0.014; Standard Deviation of all NN Intervals (SDNN): 78.3 ms ± 35.9 ms vs. 48.1 ms ± 20.5 ms, *p* = 0.001], increased HRV frequency-dependent parameter [Low Frequency (LF): *p* = 0.001], as well as reduced depressiveness (BDI-II: *p* = 0.0236). None of these parameters were changed in control patients (*p* = ns). Dizziness-associated symptoms and sympathetic function of vasculature and sweat glands were unaltered in both study arms.

**Conclusion:**

In a randomized controlled pilot study, HRV biofeedback led to improved autonomic cardiac function and alleviated symptoms of depression in patients with functional dizziness, most likely mediated by a predominantly parasympathetic effect.

## Introduction

Functional dizziness, such as persistent postural-perceptual dizziness (PPPD), is one of the most common causes of permanent dizziness and is essentially characterized by persistent vertigo without a causative peripheral vestibular, central nervous, or psychiatric disease [1, 2].

The diagnostic criteria of PPPD encompass persistent dizziness or balance disorders lasting a minimum of 3 months following a causal event (e.g., a vestibular disorder), characterized by heightened sensitivity to visual stimuli or movement. Furthermore, the symptoms have to appear at rest or during activity and substantially diminish the individual's quality of life. PPPD resembles historical non-somatic chronic vertigo concepts, including phobic postural vertigo (PPV), space-motion discomfort (SMD), visual vertigo (VV), and chronic subjective dizziness (CSD). The current understanding of PPPD remains ambiguous regarding whether it constitutes a singular disorder or represents several diseases with similar symptoms but different pathophysiological causes. Mild depressive and anxiety symptoms, along with phobic behaviors, are observed in PPV and CSD. Thus, it can be inferred that psychiatric symptoms and comorbidities, including anxiety disorders and depression, impact a considerable number of patients with PPPD, despite psychiatric disorders not being regarded as the primary cause of vertigo [3, 4].

Due to the partially high psychological strain and the related restriction of everyday life, functional dizziness is a disease with substantial socioeconomic implications [5]. The currently common and established therapeutic strategies of functional dizziness, such as PPPD include psychopharmacotherapy with selective serotonin reuptake inhibitors (SSRIs), as well as cognitive–behavioral therapy [6, 7]. Nevertheless, some patients do not respond favorably to the established therapy, leading to a considerable physical and psychological burden.

Remarkably, the majority of people experiencing vestibular symptoms show increased autonomic nervous system activity, particularly in the sympathetic nervous system. Consequently, increased sweating, increased heart rate, malaise, and dizziness are the most common symptoms reported by patients [8]. The occurrence and severity of autonomic symptoms are independent of the etiology of the vestibular dysfunction and affect both somatic and non-somatic dizziness to a similar extent. The impact of the autonomic nervous system integrity on the severity of vestibular symptoms in patients with functional dizziness and the potential therapeutic intersection is, unlike in patients with acute lesions of the vestibular system, still largely not understood and the subject of further research. However, with regard to the importance of possible psychiatric comorbidities, a sympathovagal imbalance is postulated [3, 5, 9]. HRV-biofeedback training is considered a non-invasive, non-pharmacological procedure that leads to an improvement HRV and influences neurocardiac function positively, as a result of targeted stimulation of parasympathetic efferents induced by the metronomic breathing technique [10]. A targeted neurocardiac therapy like HRV biofeedback is currently not an evidence-based part of the therapy of functional dizziness. A favorable influence of HRV biofeedback on the neurocardiac function was already proven in advance, for somatic diseases such as cardiovascular diseases as well as psychiatric diseases such as depression and anxiety disorders [11–14]. The present work evaluates the potential benefit of short-term HRV biofeedback on neurocardiac function and the alleviation of dizziness- and neuropsychiatric symptoms in therapy-naive patients with functional dizziness.

## Methods

### Study population and protocol

This two-arm study included female and male patients who were diagnosed with functional dizziness for the first time in a neurological emergency department (University Hospital Carl Gustav Carus Dresden, Saxony, Germany) following an initial presentation and extended diagnostic work-up.

We included patients between the age of 18 and 59 who had an unremarkable clinical neurological examination, caloric testing, and cranial computed tomography or magnetic resonance imaging. To reduce the impact of confounding according to the autonomic function assessment, we excluded patients with history of relevant cardiovascular diseases (diabetes mellitus, coronary heart disease, and heart failure), intake of any tricyclic antidepressant within the last 3 months, or any beta-blocker within the last 14 days.

All patients were randomized according to a previously generated allocation sequence using an online randomizer (randomizer.org) to receive six 20 min sessions of either HRV biofeedback over 2 weeks or no intervention, each without concomitant therapy. Sequentially numbered containers were used to conceal the sequence until the interventions were allocated.

At baseline, all patients underwent a detailed medical history assessment, if available, based on the information of digital medical records, and a neurological examination including evaluation for signs of peripheral-vestibular or central-nervous vertigo, as well as autonomic testing along with assessment of neurocardiac, sudomotor, and vasomotor function and psychometric testing using self-assessment questionnaires to quantify dizziness and dizziness-associated discomfort and the extent of anxiety, depressiveness, and general subjective psychological stress. In addition, the Schellong’s orthostatic test was performed to rule out severe autonomic orthostatic dysfunction causing the dizziness. The HRV biofeedback, performed with a duration of 20 min per session, took place once a day within 2 weeks, starting at baseline. Immediately after the last biofeedback session on day 14, as well as 3 and 6 weeks after baseline, autonomic function testing and psychometric assessment were repeated. The autonomic and clinical examination was carried out by an investigator who was not blinded to randomization (E.S.).

### Ethical considerations

All procedures involving human subjects/patients were approved by the institutional review board of the Technische Universität Dresden (IRB reference number: EK 1180426010). Written and oral informed consent was obtained from all patients prior to any study-related procedures.

### Study intervention

In our study, a validated HRV-biofeedback system (StressPilot™; BioSign, Ottenhofen, Germany) was used as described previously [15]. In brief, patients were instructed to breath rhythmically with a given breathing frequency (6/min). The disposed respiratory rate stimulates the cyclic oscillation of inspiratory increase and expirational reduction of heart rate most effectively, enhancing HRV [5]. A software-based biofeedback system (Stress Pilot Manager®, BITsoft Health Systems GmbH, Bitburg, Germany) with an integrated ear pulse sensor was used for HRV biofeedback training with continuous assessment and real-time visualization of HRV on the computer screen, indicated by a digital balloon moving up and down proportionally reflecting the amplitude of HRV. For this purpose, an increase and fall of the object symbolized the simultaneous change of the HRV. The HRV-biofeedback training was preceded by an oral instruction by the study physician (E.S.), who explained the breathing technique to the participants. During the biofeedback training, the breathing pace was demonstrated to the participant using a bar, that signaled inspiration and expiration through rise and fall. Each session of the HRV biofeedback training was scheduled to last 20 min. Patients in the HRV biofeedback group underwent six sessions of HRV biofeedback training over 2 weeks, with no more than one session per day. Patients in the control group did not receive HRV biofeedback training.

### Outcome parameters

Main outcome of the present study was the frequency-domain parameter root mean square of successive RR interval differences (RMSSD). Secondary outcomes comprised HRV frequency- and time-domain parameters, sudomotor and vasomotor function, as well as the degree of dizziness and dizziness-associated discomfort, anxiety, and depression.

### Assessment of autonomic functions

Testing of autonomic function was carried out after a resting period in a lying position lasting 15 min. All measurements were completed under standardized conditions with a controlled room temperature of 22 to 25 °C in an examination room with calm atmosphere. To quantify the autonomic parameters, including neurocardiac, sudomotor and vasomotor function, the biosignals were processed after derivation using an artifact filter and amplifier (Bridge Amp R FE221, ADInstruments, Castle Hill, Australia) and then digitized using a four-channel polygraph (PowerLab®, ADInstruments, Castle Hill, Australia). The analysis of the collected digital data was realized using LabChart*®* software for Windows (ADInstruments, Castle Hill, Australia).

#### Assessment of neurocardiac function

To evaluate the neurocardiac autonomic function, HRV was calculated and assessed by recording a three-channel electrocardiogram (MLA2503R ADInstruments, CastleHill, Australia). The cardiac electrical activity was registered over two phases with different breathing modes of 3 min each. In addition to a first phase, in which subjects were encouraged to breathe calmly and habitually, in the second phase, subjects were instructed to breathe deeply and at a frequency of six breathing cycles per minute to achieve an increase in parasympathetic activity [16]. An audio signal ensured the correct execution of paced breathing by indicating onset of inspiration and expiration.

Subsequently, time-domain analysis of HRV was carried out using the electrocardiograms of both measurements including normal and paced breathing. The primary outcome measure RMSSD, which is predominantly influenced by the parasympathetic nervous system was calculated, as well as the standard deviation of all normal-to-normal intervals (SDNN). The frequency-based HRV parameters total power, high frequency (HF), low frequency (LF), and very low frequency (VLF) were determined by spectral analysis using the Fast Fourier Transformation in both phases too. As an indicator for the sympathovagal balance, the ratio of low frequency to high frequency was calculated [17]. For calm and paced breathing, all HRV parameters were analyzed and reported separately.

#### Assessment of sudomotor autonomic function

The sympathetic skin response (SSR) was analyzed to evaluate sudomotor function [18]. Two finger electrodes (MLT116FR, ADInstruments, Castle Hill, Australia) attached to third and fourth finger of the non-dominant hand were used to detect the alteration in the skin conductance level caused by a sympathetic stimulus according to a sudden deep inspiration [19]. The SSR was defined as the maximum increase of amplitude of skin conductance level directly before and after the sympathetic stimulus.

#### Assessment of vasomotor autonomic function

To quantify the integrity of autonomic vasomotor function, Laser Doppler Flowmetry (LDF) was used to assess the modulation of cutaneous blood flow following sympathetic stimulation. Therefore, a plethysmograph (MLT1020PPG, ADInstruments, Castle Hill, Australia) equipped with a photoelectric sensor and attached to the distal phalanx of the index finger of the non-dominant hand was inserted, as described previously [20]. The unabsorbed infrared signal (950 nm) of a light-emitting diode reflected by the arterial, capillary, and venous erythrocyte flow at a depth of 2 mm is assumed to be proportional to the current cutaneous blood flow. During a 5-min assessment period, patients were asked to breathe casually and take a single deep breath after the first and third minute. The decrease and consecutive increase of cutaneous flow provoked by deep inspiration was evaluated to determine vasoconstrictory response and sympathetic vasomotor function. The vasoconstrictory response was defined as the difference between blood flow at baseline and the lowest value post deep inspiration over blood flow at baseline. Durations to 50% constriction and 50% redilatation of cutaneous vessels were determined as relevant time marks.

#### Psychometric measures

Severity of symptoms attributed to vertigo were assessed using the German translation of the Vertigo Symptom Scale (VSS) [21]. Perceived impairment of physical activities in everyday life, as well as the resulting restrictions on social interactions and leisure activities were assessed using the Vertigo Handicap Questionnaire (VHQ) [22]. To quantify the occurrence of relevant comorbidities, the Beck Depression Inventory II (BDI-II) was used to assess the severity of depression [23]. In addition, the Spielberger State-Trait Anxiety Inventory X1 (STAI-G X1) was used to assess anxiety [24].

Psychometric analysis of coexisting psychopathologies was carried out using the Symptom Checklist-90-Revised (SCL-90-R), a self-report instrument consisting of a 90-item questionnaire evaluating the subscales anxiety, depression, hostility, interpersonal sensitivity, obsessive–compulsive, paranoid ideation, phobic-anxiety, psychoticism, and somatization [25]. The recovery-stress questionnaire [Erholungs-Belastungsfragebogen; EBF] was used as an instrument for quantifying current subjective intensity of psychological strain [26].

### Statistical analysis

The statistical analyses were carried out with SPSS software (Version 27.0, IBM, Armonik, NY), aside from the analysis of psychometric testing, which was performed using STATA software (Version 16.1, StataCorp., College Station, TX). The change of RMSSD from post-intervention to follow-up 2 was defined as primary outcome. Additional outcomes encompassed SDNN, other time-based HRV parameters (Coefficient of Variation of NN Intervals CVNN, Average Heart Rate AHR), frequency-based HRV parameters (HF, LF, VLF, and Total Power), parameters of autonomic sudomotor (sympathetic skin response) and vasomotor (vasoconstrictory response, duration to 50% constriction, and duration to 50% redilatation) function, and results of psychometrical tests (VHQ, VSS, BDI-II, SCL-90-R, STAI-XI, and EBF-24).

Following testing for normality with Shapiro–Wilk test and equality of variances with Levene test, Student’s t test for independent samples, Wilcoxon–Mann–Whitney test, Chi-square test, and Fisher’s exact test were applied to compare baseline characteristics between groups. In case of a predominantly right-skewed distribution, the log transformation of all reported parameters was performed, so that normal distribution of the collected data may be taken for granted. To ensure a better comparability of the data, mean values and standard deviations are reported in non-logarithmic form. Exceptions to this are the presented results of the psychometric test, in which, due to the ordinal scaling, the specification of median and quartile spacing was preferred. Mixed-effects analyses of variances (ANOVA) were performed to compare main effects of time (baseline, post-intervention, follow-up 1, and follow-up 2) and study arms (intervention or control) as well as the interaction effects of time × study arms on parameters of HRV, sudomotor, and vasomotor function. Assessments at baseline, post-intervention, and follow-up 1 and 2 were considered quadruple tiered inner subject factors, while study arms were considered double-tiered between-subject factors. Additionally, main effects of examination condition (at rest and during paced breathing) were calculated for HRV parameters. Sphericity was tested with the Mauchly test. Lack of sphericity led to an adaption of the p value and the degrees of freedom according to Greenhouse–Geisser. Significant results (significant interaction, time × study arm) were subject to post hoc tests (Welch test) for study arm comparisons to allocate mean differences to distinct major effects. Bonferroni correction was conducted to adapt for multiple testing. This led to an adjustment of the significance level to *p* ≤ 0.0167. Due to the exploratory nature of this study, no formal sample size calculation was undertaken. Datasets for the primary outcome and autonomic testing were complete.

## Results

### Demographic and baseline characteristics

**Fig. 1:**
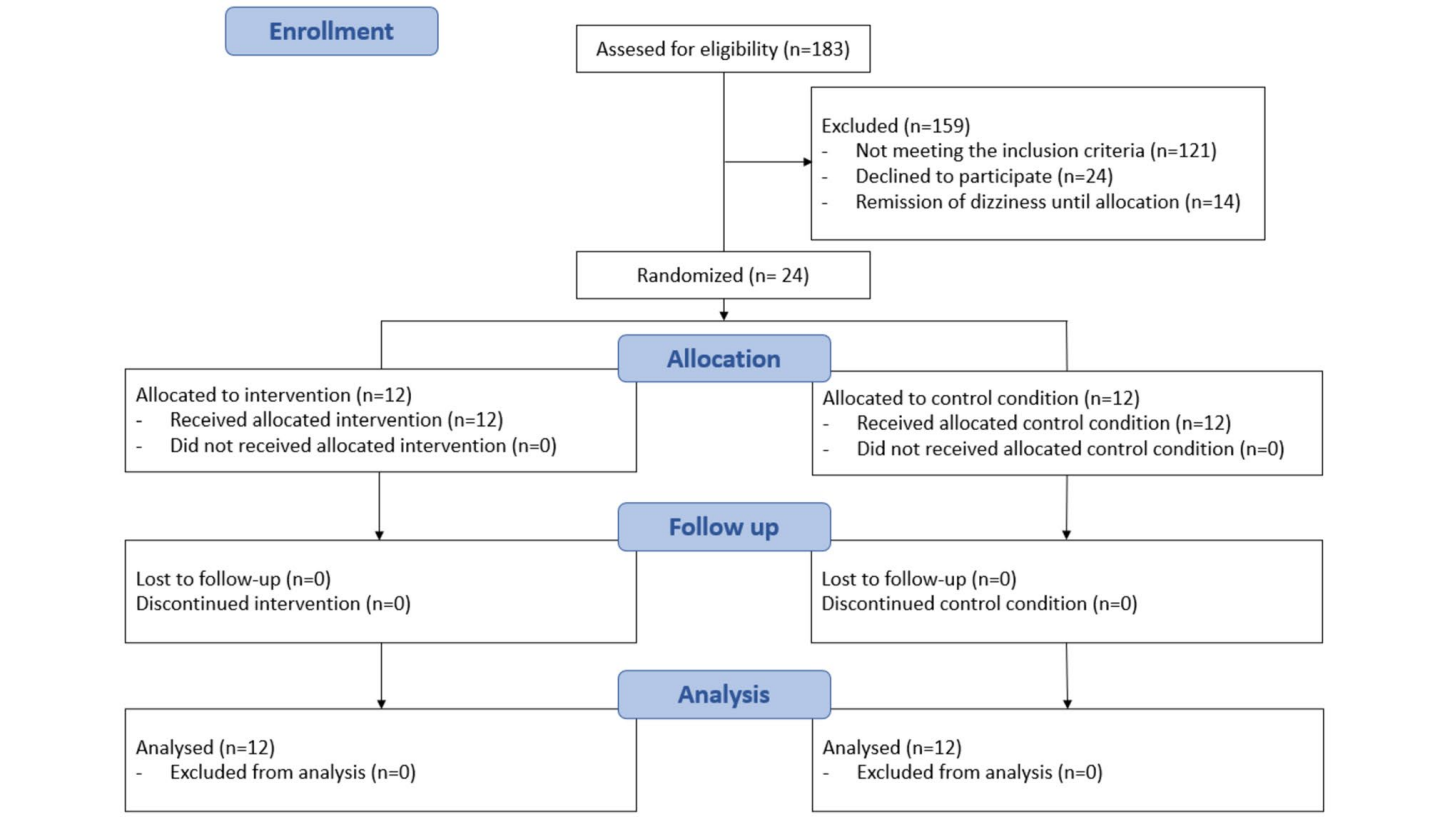
The Consolidated Standards of Reporting Trials (CONSORT) flow diagram of the progress through the phases of the parallel randomized-controlled trial conducted (modified from [27])

From 01 August 2016, to 31 July 2019, 183 patients were assessed for study eligibility. A total of 24 patients (14 males and 10 females; ages 27–54 years; 39.7 ± 9, mean ± standard deviation) with an initial diagnosis of functional dizziness agreed to participate in the study and were randomly assigned to the biofeedback (*n* = 12) and the control (*n* = 12) groups. The study flowchart is depicted in Fig. 1. Baseline characteristics, including age, sex, weight, and height, were well balanced among both study groups. Regarding comorbidities, a higher proportion of patients with migraine and arterial hypertension was observed in the control group (Table 1). A single subject included in the control group reported a concurrent mental disorder (post-traumatic stress disorder, *PTSD*).

**Table 1:** Demographic characteristics

	HRV biofeedback (n = 12)	Control (n = 12)	p value*
Demographics			
Gender, male, n (%)	8 (67)	6 (50)	
Age, years, mean ± σ	38 ± 9	40 ± 9	0.6
Weight, kg	78 ± 11	76 ± 20	0.8
Height, cm	175 ± 8	175 ± 11	0.9
Comorbidities, n (%) ^†^			
Migraine	−	3 (25)	−
Arterial hypertension	−	3 (25)	−
Smoking	−	2 (16)	−
Clinical baseline values			
Heart rate, 1/min	74 ± 9	72 ± 8	0.6
Systolic blood pressure, mmHg	130 ± 13	138 ± 18	0.9
Diastolic blood pressure, mmHg	78 ± 6	83 ± 11	0.9

Data are expressed as mean (± standard deviation) or percentage. σ, standard deviation; *kg* kilogram, *cm* centimeter, *min* minute, *mmHg* millimeter of mercury. *p value refers to between-group comparisons. ^**†**^ Further comorbidities, occurred with a prevalence of *n* = 1: (A) HRV biofeedback: bronchial asthma; chronic sinusitis; hypercholesterolaemia; irritable bowel syndrome; narcolepsy; neurodermatitis; Rolando’s epilepsy; history of cardiac ablation of AV nodal reentry tachycardia. (B) Control: chronic tinnitus; gastro-oesophageal reflux disease; hypothyroidism; post-traumatic stress disorder; history of benign paroxysmal positional vertigo; history of left cerebral artery infarction; history of right vertebral dissection

### Neurocardiac function

**Fig. 2:**
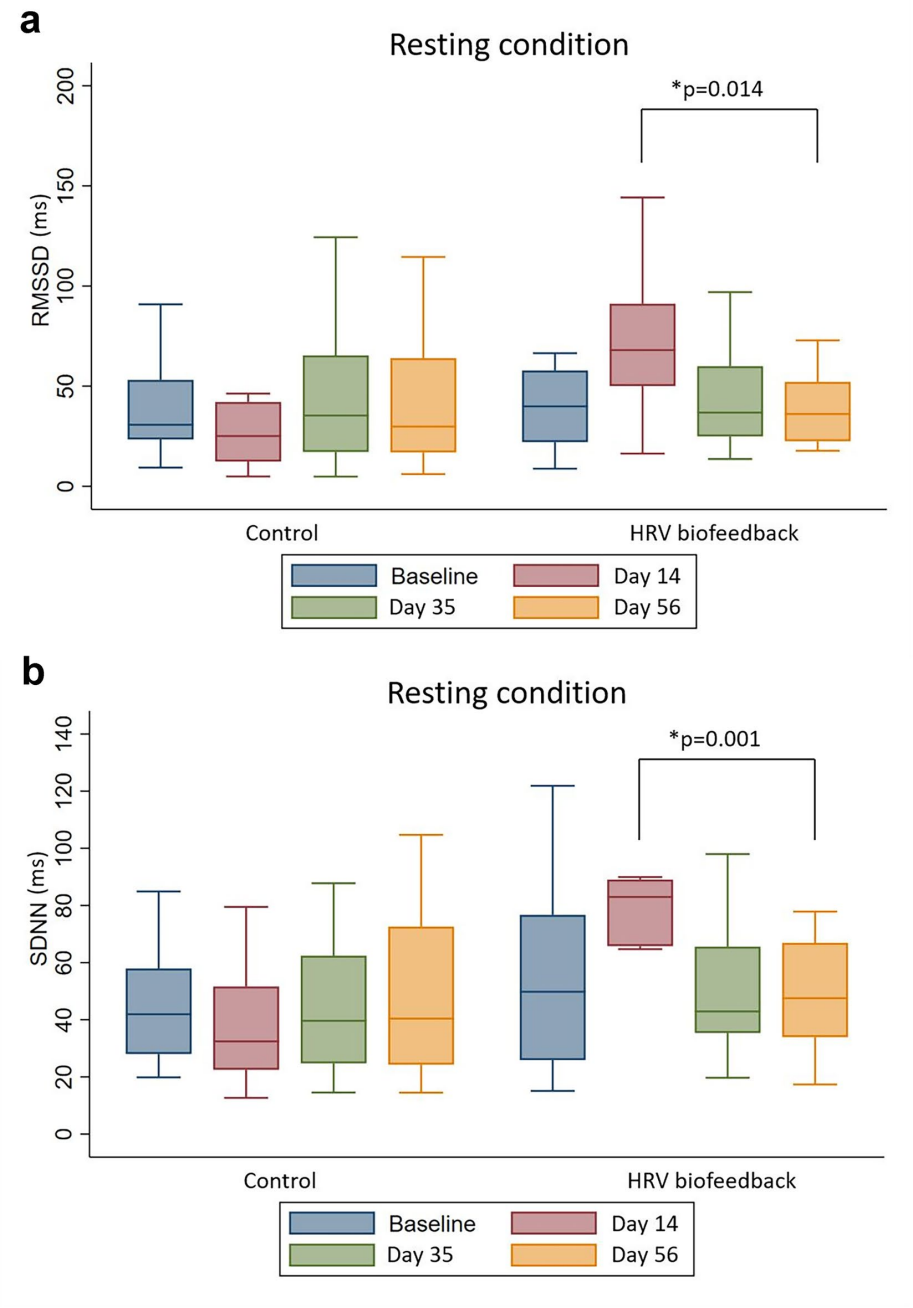
The Box-and-Whisker plot illustrates an increase in heart rate variability assessed via **a** RMSSD and **b** SDNN under resting condition after HRV biofeedback (*n* = 12) but not within the control group (*n* = 12). The mean is depicted as horizontal line. *p value refers to pair comparisons in selected timexgroup interactions determined using analysis of variance with repeated measures

Patients who received HRV biofeedback displayed an increase in HRV under normal breathing condition with elevation of predominantly parasympathetic HRV measure RMSSD (Fig. 2a) post-intervention compared to follow-up 2 [0.56, 95%CI (0.1,1.01); *p* = 0,014], which was not observed in the control group. The time-domain analysis of HRV with SDNN was also elevated in the treatment group post-intervention compared to follow-up 2 [0.45, 95%CI (0.2, 0.69); *p* = 0,001] after HRV biofeedback which was neither evident in the control group (Fig. 2b). Furthermore, spectral analysis of HRV under casual breathing showed an increase in LF immediately post-intervention compared to follow-up 1 [1,071, 95%CI (0,169,1,972); *p* = 0.017] and follow-up 2 [0.935, 95%CI (0.425,1.446); *p* = 0.001], but not for participants in the control group (Table 2).

**Table 2:** Measures of autonomic cardiac, sudomotor, and vasomotor function

Parameter	Resting condition		Paced breathing	
	HRV biofeedback (*n* = 12)	Control (*n* = 12)	HRV biofeedback (*n* = 12)	Control (*n* = 12)
Baseline				
High frequency (ms^2^)	2713 ± 5808	985 ± 1391	985 ± 1391	1797 ± 1591
Low frequency (ms^2^)	1511 ± 1892	1387 ± 2361	5988 ± 5106	6506 ± 4529
Total power (ms^2^)	6128 ± 10,615	4718 ± 9730	8922 ± 7254	9663 ± 6854
Sympathetic skin response (µs)	2.78 ± 2.35	3.27 ± 2.82	−	−
Vasoconstrictory response (%)	0.0016 ± .002	0.0016 ± .0016	−	−
Postintervention				
High frequency (ms^2^)	2097 ± 2444	605 ± 829	2450 ± 3291	2080 ± 4876
Low frequency (ms^2^)	1854 ± 1117 *	403 ± 374	7619 ± 5649	5512 ± 6819
Total power (ms^2^)	5997 ± 4370	1748 ± 1930	11,995 ± 9395	9951 ± 17,010
Sympathetic skin response (µs)	1.71 ± 1.59	2.49 ± 2.11	−	−
Vasoconstrictory response (%)	0.0015 ± .0019	0.0019 ± .0018	–	−
Follow-up 1				
High frequency (ms^2^)	729 ± 814	1035 ± 1288	996 ± 991	2094 ± 2585
Low frequency (ms^2^)	760 ± 880	848 ± 967	5730 ± 4637	8235 ± 6289
Total power (ms^2^)	2685 ± 2524	2782 ± 2883	7749 ± 5443	11,442 ± 8955
Sympathetic skin response (µs)	2.57 ± 2.33	2.12 ± 1.6	–	–
Vasoconstrictory response (%)	0.0019 ± .0019	0.0015 ± .0013	–	–
Follow-up 2				
High frequency (ms^2^)	598 ± 529	1169 ± 2373	926 ± 1225	1660 ± 2771
Low frequency (ms^2^)	785 ± 752 *	838 ± 953	5297 ± 4182	5516 ± 4606
Total power (ms^2^)	2470 ± 1826	7426 ± 5902	2642 ± 3127	8220 ± 7868
Sympathetic skin response (µs)	1.83 ± 1.8	2.81 ± 3.67	–	–
Vasoconstrictory response (%)	0.0019 ± .0016	0.0013 ± .0012	–	–

Data are expressed as mean (± standard deviation). *Ms* milliseconds, *µS* microsievert. ^*^Significant pair comparisons in selected timexgroup interactions determined using analysis of variance with repeated measures, *p* value < 0.01; other *p*-values = ns

A tendency toward statistical significance and numerical increase was found under analysis of HRV in non-rhythmic breathing for the frequency-based parameters HF [F (2.02,44.5) = 3.06; *p* = 0.056] and Total Power [F (1.9,43.29) = 3.04; *p* = 0.059] postinterventionally compared to follow-up 2 (Table 2).

Other parameters of spectral analysis (LF, HF, and total power) and on time-domain analysis (CVNN, AHR, and SD of ΔNN) assessed under resting conditions remained unchanged in both groups. Regarding the variation of autonomic cardiac function under paced breathing, no difference could be detected between the two study groups, neither for frequency-based parameters nor for time-based parameters.

### Sudomotor and vasomotor autonomic function

Assessment of autonomic sudomotor function by measuring the SSR in patients with functional diziness revealed no changed either after HRV biofeedback training or within the control group (Table 2).

In addition, the quantification of autonomic vasomotor function using laser Doppler flowmetry of cutaneous blood flow upon sympathetic stimulation did not show any differences between the two study arms and remained without any relevant impact of HRV-biofeedback training (Table 2).

### Symptoms of PPPD

We observed a decrease in vertigo-associated burden measured by the VHQ both in the biofeedback group [Q (3) = 9.1565, *p* = 0.027] and in the control group [Q (3) = 14.4872, *p* = 0.002]. The severity of vertigo symptoms, quantified by VSS, remained unaltered in both study arms (Table 3).

**Table 3:** Psychometric evaluation

Intervention	Time	Scale					
		VHQ	VSS	BDI-II	STAI-XI	EBF-24	SCL-90
HRV biofeedback (*n* = 12)	Baseline	42.5 (26)	32.5 (16.5)	11.5 (7.5)	38.5 (14)	75.5 (30.5)	51.5 (61)
	Postintervention	36 (28.5) *	24 (18)	8 (9) *	36.5 (12)	90 (14)	41.5 (58.5) *
	Follow-up 1	34 (25.5) *	27 (28)	7 (11.5) *	34 (14.5)	93.5 (31)	32.5 (37) *
	Follow-up 2	32 (29) *	22.5 (24)	6 (9) *	32.5 (12)	98.5 (29.5)	31 (51.5) *
Control (*n* = 12)	Baseline	46 (24.5)	30.5 (14.5)	9.5 (13.5)	39.5 (14)	76 (25)	53.5 (58.5)
	Postintervention	37.5 (23) *	21 (18.5)	9 (10.5)	35 (6.5)	84 (17)	41.5 (76) *
	Follow-up 1	33.5 (27) *	23.5 (17)	7.5 (14)	40.5 (13.5)	79.5 (17.5)	39.5 (55) *
	Follow-up 2	33.5 (32) *	21.5 (18)	2.5 (14.5)	36 (8.5)	85 (25.5)	25.5 (80) *

Data are expressed as median (interquartile range). *Significant main effect over time determined using analysis of variance with repeated measures, *p* value < 0.05. *VHQ* vertigo handicap questionnaire, *VSS* vertigo symptom scale, *BDI-II *Beck-depression-inventory-II, *STAI-X1 *State-trait-anxiety-inventory-X1, *EBF-24* “Erholungs-Belastungsfragebogen-24”, *SCL-90* symptom-check-list

### Depression and anxiety

The use of HRV biofeedback led to an alleviation of severity of depressive symptoms [Q (3) = 9.4706, *p* = 0.024], which was not observed within the control group [Q (3) = 5 0.6389, *p* = 0.131]. In the HRV-biofeedback group, anxiety assessed using the STAI-XI showed a numerical decrease and tendency toward statistical significance [Q (3) = 7.4727; *p* = 0.058], in contrast to the control group [Q (3) = 2.6348, *p* = 0.451]. During analysis of the results of the SCL-90-R, a decrease of severity of psychiatric symptoms in the intervention group [Q (3) = 10.6271, *p* = 0.014] as well as the control group [Q (3) = 8.0256, *p* = 0.046]. Regarding the EBF, no effect was observed in either the intervention group or the control group (Table 3).

## Discussion

The main results of our study were that after a 2-week program of six HRV biofeedback sessions, parasympathetic HRV measures were increased compared to the sham intervention in patients with functional dizziness. This observation might be indicative of a beneficial effect of the treatment on autonomic neurocardiac function. This interpretation has to be considered hypothesis-generating because of its explorative design and small sample size. It is noteworthy that this potentially beneficial effect was most pronounced immediately post-interventional and diminished during the subsequent observation period. Second, sympathetic measures of autonomic function showed no differences between the two study groups. Third, we observed an alleviation of the severity of symptoms of depression after HRV biofeedback compared with the sham intervention. Finally, the treatment was well tolerated. Taken together, our study provides pilot data that may be consistent with beneficial effects of HRV biofeedback in patients with functional dizziness and may form a basis for further investigation in larger study populations.

Chronic functional vestibular disorders such as PPPD are associated with a variety of psychiatric symptoms, with depression and anxiety being the most common [28]. Individuals with PPPD exhibit higher levels of anxiety in comparison to those with other vestibular disorders; however, the intensity of depression remains nearly equal. Nevertheless, patients with PPPD had elevated levels of depression and anxiety when compared prospectively to those who recovered from vestibular symptoms. The severity of depression and anxiety is associated with the duration of the disease. It is uncertain if persons with pre-existing mood and anxiety disorders are at an elevated risk of developing PPPD, or if those with PPPD later have secondary mood and anxiety disorders [29, 30].

Establishing causation between psychiatric diseases and PPPD is challenging, as individuals with depression, generalized anxiety disorder, agoraphobia, social phobia, obsessive–compulsive disorder, and trauma-related conditions may have chronic dizziness without fulfilling the criteria for PPPD. Moreover, traumas and other negative life experiences are equally prevalent among individuals with structural vestibular disorders [1].

A number of randomized-controlled trials or pilot studies in conditions associated with reduced HRV, such as coronary heart disease, chronic obstructive pulmonary disease, stroke, depression, anxiety disorders, and alcohol dependence, have shown that HRV biofeedback improves clinical outcomes in cardiovascular and neuropsychiatric comorbidities [11, 12, 31–34]. HRV biofeedback is understood to be based on the physiological phenomena of respiratory sinus arrhythmia. Heart rate rises during inspiration and falls during expiration, and vice versa. Baroreflex activation causes a parasympathetically mediated reduction in heart rate by rhythmic breathing at a specific pace [16].

Generally, an imbalance of the dichotomous autonomic nervous system toward a chronic increased sympathetic tone or a permanently reduced parasympathetic tone is postulated to be present in psychiatric diseases, such as depression, post-traumatic stress disorder (PTSD), and alcohol addiction [31, 35, 36]. The coexistence of vertigo sensations and autonomic dysregulation has also been reported in the context of vestibular diseases. This is remarkable due to apparent vegetative symptoms, such as increased sweating, elevated pulse rate, and malaise. The incidence and extent of the accompanying vegetative symptoms seems to be independent of the etiology of the vestibular dysfunction and affects both somatic and non-somatic vertigo symptoms [3, 37, 38].

Motion sickness is another example of the relationship between increased sympathetic nervous system activity and vestibular stimulation. It has been demonstrated that vestibular stimulation reduces baroreflex activation in this condition [39].

Our findings indicate an increase in the time-domain parameters RMSSD and SDNN, as well as a trend toward higher HF and total power, which are consistent with an autonomic nervous system's primarily parasympathetic regulation of neurocardiac control [17]. Furthermore, the change in total power estimated by spectral analysis implies an overall rise in HRV. An increase in LF, which is expected to be influenced by both parts of the autonomic nervous system, and changes in the time-analyzed HRV parameters during calm breathing but not parasympathetic stimulating paced breathing may indicate a modulation of baroreflex sensitivity and a sympathetic effect of HRV biofeedback.

The formerly reported higher accuracy of time-domain analysis over spectral analysis of frequency domains in neurocardiac function evaluation could support these results [40], especially in combination with the noted increase in SDNN and RMSSD after HRV biofeedback. This conclusion is also consistent with the ongoing controversy concerning the diagnostic utility of spectral analysis, which has been questioned due to growing evidence of the technique's low accuracy in discriminating between sympathetic and parasympathetic HRV components [41].

The significant inter-individual variability and habituation of sympathetic skin response (SSR) techniques used to evaluate skin conductance might partially explain the lack of modulation on sudomotor function. Alternative measurement methods with lower inter-individual variability, such as the "Quantitative Sudomotor Axon Reflex Test" (QSART) and the "Quantitative Direct and Indirect Test of Sudomotor Function (QDIRT)," are, in our view, limited in their applicability due to more complex clinical procedures and the feasibility of potential follow-up studies [42]. On the other hand, the lack of changes in SSR following HRV biofeedback might lend support to a primarily parasympathetic mechanism whereby HRV biofeedback alters neurocardiac function as previously suggested [10, 34].

While we observed changes on predominantly parasympathetic measures such as RMSSD, we did not note any modulation of HRV following non-metronomic calm breathing. Paced breathing was assessed as a secondary analysis to characterize HRV under enhanced parasympathetic outflow. However, this was an explorative additive analysis. Using this technique, we recently showed additional value in the assessment of neurocardiac functional integrity after HRV biofeedback in patients with critical illness neuropathy [43]. This suggests an additional sympathetic component underlying HRV biofeedback's neurocardiac function modulation.

Furthermore, the lack of change in time and frequency analysis parameters during the parasympathetic stimulation maneuver could be due to a combination of several aspects. Potential confounders such as diseases associated with altered cardiac autonomic function and reduced HRV (coronary heart disease, diabetes mellitus, depression, or schizophrenia) or pharmaceutical influences (beta-receptor blockers or tricyclic antidepressants) did not occur [44, 45]. The instructed breathing frequency was sufficiently internalized by the participants. Thus, they learned to breathe spontaneously with an HRV-increasing resonance frequency during the intervention. They might have kept and carried on this skill beyond the duration of the interventional protocol. The learning effects may have reduced baseline differences in individuals who received HRV biofeedback training in the active study arm.

In addition, the sample of young, somatically unimpaired individuals may have a minor baseline HRV decrease. A comparison of the mean values of the parameters RMSSD, HF, LF, and Total Power obtained in this study with common standard values acquired from several studies with a small number of cases reveals a partial increase among these variables [17]. As a result, it turns out that the effect of synchronized breathing on stimulation of parasympathetic system was already restricted in calm breathing due to quite modest reduction of HRV. Paced breathing would cause a vagal stimulation, but the small sample size and increased baseline HRV would render it ineffective to be significant.

The observed increase in RMSSD indicates a post-interventional parasympathetic modulation of heart rate variability that diminishes over the observation period. This aligns with previous research on HRV biofeedback in other study populations, such as patients with alcohol dependence [46], and indicates that only a transient improvement of parasympathetic modulation can be achieved by six training sessions of HRV biofeedback. Increased exercise frequency and duration may support the maintenance of parasympathetic function. Alternative therapy modalities, such as a mobile biofeedback application, necessitate evaluation to determine their potential in enhancing the feasibility of continuous biofeedback therapy.

Despite a high proportion of physically healthy individuals, the baseline characteristics of the study population revealed an uneven distribution of comorbidities, notably migraine and arterial hypertension. Migraine, a prevalent neurological disorder, shows a complex bidirectional relationship with psychiatric comorbidities, including major depression, panic disorder, and stress-related disorders [47]. The absence of diagnosed psychiatric disorders in migraine patients at the time of study inclusion does not fully reduce the potential impact of the random unequal distribution of individuals with migraine between the two study groups on the outcomes and efficacy of the investigated intervention. This may also be important considering that the treatment of the different migraine comorbidities requires different pharmacologic and non-pharmacologic approaches. The influence of these factors on the results and the assessment of the investigated therapy's efficacy remains uncertain, particularly due to the absence of a comprehensive classification of potential psychiatric comorbidities among the studied patients. Currently, there is no evidence, indicating that the study intervention affects the progression of non-vestibular symptoms in migraine patients [48].

All studies were conducted as outpatient examinations. This may lead to the exclusion of certain populations with severe symptoms. It may clarify the comparatively mild restriction of autonomic function parameters in comparison with the results of other observations using the same approach in different somatically or psychiatrically impaired populations [34, 46]. However, since the examinations are conducted in an outpatient setting, the results tend to indicate the effectiveness of biofeedback training in relation to actual clinical practice and can thus be viewed as a suggestion of external validity. The majority of patients were recruited after being diagnosed with functional dizziness for the first time during an emergency appointment to a neurological outpatient department. Therefore, impacts of additional treatment techniques, such as cognitive–behavioral therapy, on psychometric variables and autonomic functions are limited [49].

This study suggests that HRV biofeedback may improve neurocardiac function in patients with functional dizziness. Due to its feasibility, it may be possible to integrate the treatment into a standardized multimodal therapy setting such as vestibular rehabilitation [50]. Furthermore, a recent review concluded that a multimodal treatment strategy involving behavioral therapy, vestibular rehabilitation, and SSRI medication proved highly beneficial, particularly when compared to individuals with exclusively somatic diagnosis [51]. However, there are presently no clear recommendations for a treatment that addresses the individual subgroups of functional dizziness in the absence of an appropriate number of prospective trials. Currently, there is a lack of study-based evidence for the use of additional therapy approaches such as biofeedback approaches.

The observed reduction in depressive symptoms in the intervention group, as assessed by the BDI-II, is consistent with the results of other studies in study populations with varying degrees of depression, although the influence of alternative therapeutic procedures on the observed results was largely minimized by our study design [14]. Overall, the observed benefits are consistent with previous HRV biofeedback research in patient groups diagnosed with major depression, alcohol dependence, or anxiety disorders [31, 45]. This also seems reasonable considering that psychiatric comorbidities such as depression or anxiety and panic disorders have been reported in a significant proportion of patients with PPPD. Furthermore, recent experimental techniques to modify cardiac autonomic function, such as non-invasive vagus nerve stimulation, have shown short-term reductions in the severity of dizziness and associated affective symptoms [52]. In addition to the reduction in BDI-II scores in the intervention group, VSS, VHQ, STAI-G X1, and SCL-90-R outcomes decreased in both groups. Since all patients were treatment-naïve and received no diagnostic assessment before initial presentation, the patient care during the trial and consideration of alternative treatment options may have improved psychiatric symptoms. All patients were screened in a neurological emergency room; therefore, symptoms may have been exacerbated upon study inclusion. The time period between the acute incident and the intervention may have relieved mental symptoms regardless of the intervention. However, the intervention group's BDI-II lessening of depressive symptoms and STAI-XI numerical decline and trend toward statistical significance may imply an additional benefit.

We observed alleviated symptoms of depression after HRV biofeedback that were sustained at the second follow-up. While the small sample size requires careful consideration and interpretation of this result, a previous study on HRV biofeedback training in individuals with Major Depression indicated a comparable inconsistency between neurocardiac function and psychiatric symptoms [31].

A proposed explanation for the separation of therapeutic effects is that as depressive symptoms decrease via therapy, the vagus nerve may no longer be activated to sustain clinical benefits, despite the internalization of regular rhythmic breathing. Second, an increased and positive selfperception during HRV biofeedback training may enhance self-efficacy, mindfulness, and cognitive and behavioral modifications, thereby explaining the enduring impact on psychometric measures beyond HRV.

Despite a rather high between-subject variability and a limited sample size, we observed an improvement in both cardio-autonomic function and affective symptoms post-intervention, which was not seen after non-intervention. It remains to be investigated whether this effect can be reproduced in a larger study population and whether the resulting higher power will indicate that the improvement in cardio-autonomic function could also translate into an improvement in affective symptoms and vestibular complaints. In addition, our data provide a basis for assessing whether a more intensive treatment protocol with increased frequency and duration of HRV biofeedback benefits symptoms of psychiatric comorbidities.

Although the differences in time-domain HRV measures between patients treated with HRV biofeedback and the control group were small, they may be clinically significant, because reduced HRV has been associated with an increased risk of death and cardiovascular events, reinforcing the importance of HRV biofeedback therapy as a potential additive treatment modality [53, 54]. Again, it should be noted that due to the pilot nature of our study, confirmation of this result by a larger population is required before a definite recommendation can be derived.

Strengths of our study include the randomized control design and the measurement of non-cardiac vasomotor and sudomotor sympathetic function parameters.

The small number of cases and the absence of a sham intervention as well as the lack of a long-term follow-up assessment of neurocardiac function are to be identified as limitations of the present work and do not allow the generalization of study data. We do not know if the observed increase of HRV after treatment would be sustained in later phases of therapy. Nevertheless, we noticed a steady decrease in time- and frequency-analytical parameters through the observation period of the follow-up consultations. More clinical testing is needed to investigate whether extending the treatment period may provide long-term maintenance of this beneficial therapeutic effect.

## Conclusion

This study provides pilot data in favor of the hypothesis that HRV biofeedback may be a useful supplementary treatment for the treatment of functional dizziness. Our observation may be helpful to the design of future confirmative multicenter trials in larger study populations.

## Data Availability

The datasets used and/or analyzed during the current study are available from the corresponding author on reasonable request.
